# Serum secreted phosphoprotein 1 level is associated with plaque vulnerability in patients with coronary artery disease

**DOI:** 10.3389/fimmu.2024.1285813

**Published:** 2024-02-15

**Authors:** Ke Huang, Shuai Chen, Lin-Jun Yu, Zhi-Ming Wu, Qiu-Jing Chen, Xiao-Qun Wang, Fei-Fei Li, Jing-Meng Liu, Yi-Xuan Wang, Lin-Shuang Mao, Wei-Feng Shen, Rui-Yan Zhang, Ying Shen, Lin Lu, Yang Dai, Feng-Hua Ding

**Affiliations:** ^1^Department of Vascular and Cardiology, Rui Jin Hospital Shanghai Jiaotong University School of Medicine, Shanghai, China; ^2^Institute of Cardiovascular Diseases, Shanghai Jiaotong University, School of Medicine, Shanghai, China; ^3^Shanghai Clinical Research Center for Interventional Medicine, Shanghai, China

**Keywords:** atherosclerosis, vulnerable plaque, single-cell RNA sequencing, SPP1, data integration

## Abstract

**Background:**

Vulnerable plaque was associated with recurrent cardiovascular events. This study was designed to explore predictive biomarkers of vulnerable plaque in patients with coronary artery disease.

**Methods:**

To reveal the phenotype-associated cell type in the development of vulnerable plaque and to identify hub gene for pathological process, we combined single-cell RNA and bulk RNA sequencing datasets of human atherosclerotic plaques using Single-Cell Identification of Subpopulations with Bulk Sample Phenotype Correlation (Scissor) and Weighted gene co-expression network analysis (WGCNA). We also validated our results in an independent cohort of patients by using intravascular ultrasound during coronary angiography.

**Results:**

Macrophages were found to be strongly correlated with plaque vulnerability while vascular smooth muscle cell (VSMC), fibrochondrocyte (FC) and intermediate cell state (ICS) clusters were negatively associated with unstable plaque. Weighted gene co-expression network analysis showed that Secreted Phosphoprotein 1 (SPP1) in the turquoise module was highly correlated with both the gene module and the clinical traits. In a total of 593 patients, serum levels of SPP1 were significantly higher in patients with vulnerable plaques than those with stable plaque (113.21 [73.65 - 147.70] ng/ml versus 71.08 [20.64 - 135.68] ng/ml; *P* < 0.001). Adjusted multivariate regression analysis revealed that serum SPP1 was an independent determinant of the presence of vulnerable plaque. Receiver operating characteristic curve analysis indicated that the area under the curve was 0.737 (95% CI 0.697 - 0.773; *P* < 0.001) for adding serum SPP1 in predicting of vulnerable plaques.

**Conclusion:**

Elevated serum SPP1 levels confer an increased risk for plaque vulnerability in patients with coronary artery disease.

## 1 Introduction

Acute coronary syndrome (ACS) is a rapidly progressing and life-threatening disease characterized by a sudden reduction in myocardial blood supply, most commonly due to rupture of atherosclerotic plaque with subsequent thrombus formation (1). Although well-developed techniques such as percutaneous coronary intervention allow quick and efficient blood flow restoration in culprit vessels, they do little to prevent future cardiovascular events (2). Pathological autopsy results have described the characteristics of such rupture-prone plaques in patients with sudden cardiovascular death (3). Recent clinical trials have suggested that patients with such unstable plaque were at increased risk for future major adverse cardiac events (MACEs) (4, 5). Therefore, early detection of the so-called unstable plaque—vulnerable plaque should be given the top priority. Recently, vulnerable plaque has been commonly summarized as a type of plaque with one of the following features such as a thin cap with large lipid core, active inflammation, severe stenosis and superficial calcified nodules (6–8), which could be detected by diagnostic imaging techniques such as intravascular ultrasound (IVUS).To date, only few biochemical markers have been identified to play a predictive role in vulnerable plaque, thus limiting their importance in clinical practice.

Secreted phosphoprotein 1 (SPP1), also known as osteopontin, was initially identified as sialoproteins deriving from bone matrix (9), with presumed involvement in bone morphogenesis and calcification. As a member of the small integrin-binding N-linked glycoprotein (SIBLING) family, SPP1 can serve as both cell adhesion modulators and cytokines through their interaction with cell surface receptors. Purified SPP1 injection in rat led to an increase in macrophage infiltration (10), while the obstructed kidney model of SPP1 null mice exhibited a significant reduction in acute macrophage infiltration (11), suggesting its regulatory role in inflammation in several inflammatory diseases, including atherosclerosis (12). Clinically, Elevated serum SPP1 levels have been linked to the presence and severity of coronary artery disease (13). More recently, several studies have identified that high SPP1 levels could also act as a novel biomarker for predicting major adverse cardiac events in patients with atherosclerotic cardiovascular disease (14–17). These findings suggest that SPP1 may contribute to the development of atherosclerotic plaque and potentially be associated with plaque vulnerability.

Single-cell sequencing is one of the most powerful techniques for dissecting cellular networks. By mapping cells in tissues or organs, it clarifies the molecular regulatory patterns and state changes of cells, providing systematic insights into the cellular interaction networks at single-cell resolution. Numerous studies are now focusing on constructing and mapping the plaque landscape at the single-cell level (18–20), which allows further understanding of the cell-cell communication network in the plaque. However, its high cost limits its role in clinical research, which could be perfectly complemented with traditional RNA sequencing. The integration of bulk RNA and single cell sequencing data provides a further step to improve our understanding of cross-talks between cells in the plaque to explore significant hub genes, which could be performed using bioinformatics tools such as Scissor (21) or WGCNA (22).

The purpose of the study was to identify susceptible cell type and hub genes associated with the formation of vulnerable plaque by combining single-cell and bulk transcriptome data with bioinformatics tools and to provide novel non-invasive biomarkers by analyzing serum levels of candidate genes from patients with coronary plaques. The study commenced with an initial analysis of omics data, subsequently progressing to clinical validation of our omics results.

## 2 Methods

### 2.1 Study population

A total of 1751 consecutive patients with coronary artery disease referred for diagnostic coronary angiography and IVUS from January 2020 to December 2022 were enrolled from the database of the Shanghai Ruijin Hospital Percutaneous Coronary Intervention Outcomes Program. Diagnosis criteria of coronary artery disease, hypertension and diabetes were consistent with our previous studies (23, 24). For this research, patients with acute coronary syndrome (n = 405), chronic total occlusion (n = 487), a history of coronary revascularization (n = 127), malignant tumor or immune system disorders (n = 78), chronic kidney diseases requiring hemodialysis (n = 14) were excluded. Forty two patients were further excluded due to unavailability of blood sample. Patients with not available IVUS image data were also excluded (n = 5). Thus, the remaining 593 patients were included in the final analyses. Baseline demographics, risk factors for coronary artery disease, and medications of all patients were recorded. This analysis was approved by Ethics Committee of the Ruijin Hospital and Shanghai Jiao Tong University School of Medicine (RJH20140311), and written informed consent was obtained from all patients.

### 2.2 Coronary angiography

Coronary angiography was performed through radial or femoral approach. Quantitative coronary angiography was performed using the Cardiovascular Measurement System version 3.0 software (Terra, GE, USA) by two interventional cardiologists. Significant coronary artery disease was diagnosed if luminal diameter narrowing was estimated as ≥ 50% in a major epicardial coronary artery. The SYNTAX score and Gensini score were calculated and used as indices of the anatomic extension and severity of coronary atherosclerosis (25, 26).

### 2.3 Intravascular ultrasound

IVUS imaging was performed after intracoronary administration of nitroglycerin (200 μg) with a motorized transducer pullback system and a commercial scanner (Galaxy; Boston Scientific, Natick, Massachusetts, USA) consisting of a rotating 30 or 40-MHz transducer. Imaging results were acquired from beyond the target lesion and rendered with a motorized catheter pullback system set at a speed of 0.5 mm/s. All real-time images were recorded on a disk for subsequent analysis. IVUS imaging analysis was performed by three independent analysts according to the criteria of American College of Cardiology Clinical Expert Consensus Document on IVUS (27). Vulnerable plaque was defined by plaque rupture or hypoechoic plaque with at least one of the following based on previous research with minor modifications (28–31) 1) attenuated plaque, 2) microcalcification (lesions of 1 to 4 mm in length and < 90° arc of calcification), 3) thrombosis 4) thin-cap fibroatheroma (TCFA). Using planimetry software (Virtue intra-Vascular imaging, Beijing, China), external elastic membrane (EEM), stent, and lumen cross-sectional area (CSA) were determined. Plaque burden was calculated as the ratio of plaque CSA (EEM minus lumen CSA) to EEM.

### 2.4 Sample acquisition and biochemical measurement

Blood samples were obtained from patients undergoing angiography after 12 hours of fasting. Samples were collected by centrifugation at the speed of 3000rpm for 10 minutes. All serum samples were stored at -80°C until analysis. Serum glucose, glycosylated hemoglobin A1c (HbA1c), blood urea nitrogen, creatinine, uric acid, and lipid profiles were measured with standard laboratory techniques on a Hitachi 912 Analyzer (Roche Diagnostics, Germany). The modified estimated glomerular filtration rate (eGFR) was calculated. Serum Secreted Phosphoprotein 1(SPP1) level were assayed with commercially available ELISA kits (SX01123, Shanghai Senxiong Technology Industrial Co.) according to the manufacturer’s instructions. The absorbance value at 450m was checked with a microplate reader, and the final SPP1 level was presented in ng/ml with a small inter-assay variations (<10%).

### 2.5 Single cell RNA sequencing data collection and integration

The single-cell transcriptome data containing three human plaque samples were obtained from the GEO dataset (GSM4705589-GSM4705591 from GSE155514). The zip files with expression matrix, features and barcodes files were downloaded and performed with Read10x Function using Seurat (32), Standard preprocessing was performed to obtain nFeature count and percentage of mitochondrial RNA for quality control. Then, we integrated three samples using the R package Harmony as previously described (33). Next, we used Seurat functions such as “RunPCA”, “FindNeighbors”, “FindClusters” and “FindAllMarkers” to identify different cell populations. Finally, we annotated the cell clusters by R package SingleR (34) and markers provided in the previous research (19).

### 2.6 Identification of phenotype-associated cell cluster

Single-Cell Identification of Subpopulations with Bulk Sample Phenotype Correlation (Scissor) was developed by Xia’s lab to identify novel cell subpopulations with a given phenotype from single-cell and RNA-seq data based on a similarity measure and computation of a cell–cell similarity network (21). Briefly, through the integration of a bulk RNA expression matrix with phenotype data and a single-cell RNA sequencing dataset, we were able to identify the cell subpopulations most strongly associated with previously unreported information in the single RNA-sequencing dataset. Scissor positive (Scissor+) cells and Scissor negative (Scissor−) cells were identified by the algorithm as positively and negatively associated with the phenotype of interest, respectively. Therefore, we downloaded RNA-seq data with corresponding clinical information from the GEO dataset (GSE120521) (35). The GSE120521 dataset contained unstable and stable plaque sections from 8 patients. By inputting the single cell expression matrix (GSE155514), bulk expression matrix and clinical information as mentioned above, we were able to define cell subsets that were the most highly relevant to vulnerable plaque phenotype.

### 2.7 Differentially expressed genes identification and functional enrichment analysis

We performed differential expression analysis between the most highly relevant cell subsets detected in single-cell transcriptome dataset using the Seurat “FindMarkers” function. The gene with absolute log_2_FC threshold ≥1.25, difference ≥ 0.2 (difference in the percentage of two cell clusters) and *P* value <0.05 was considered as a hub gene. The volcano plots were also generated to compare the expression levels of differential genes between groups using ggplot2 in R. Next, through the ClusterProfiler package, Gene Ontology (GO) and Kyoto Encyclopedia of Genes and Genomes (KEGG) pathway enrichment analyses of these hub genes were carried out as previously described to investigate their potential roles in this status (36). The results were generated using ggplot2 as bar plots or others.

### 2.8 Cell communication analysis

The CellChat package was utilized to infer, analyze, and visualize cell-cell communication between phenotype-associated cell clusters and others (37). The ligand-receptor interaction database was included in the package. All analyses were performed according to the official workflow.

### 2.9 Weighted gene correlation network analysis

The Weighted gene correlation network analysis (WGCNA) package (22) was used to construct the co-expression network and uncover the correlation of genes and critical interacted genetic modules based on the microarray expression matrix (GSE28829) (38). The Soft thresholding power β was selected when the fit index of scale-free topology first reached 0.90 using PickSoftThreshold function. Co-expression modules were then established via dendrogram. The Pearson correlation of each module’s eigengene with phenotypes was analyzed and shown in the module-trait heatmap. We then selected the most correlated module and evaluated genes in the module to identify hub genes with gene significance>0.7 and module membership>0.8. Module membership represents the relationships between gene expression profiles and module eigengenes, and gene significance represents the absolute value of the associations between gene expression and module traits.

### 2.10 Statistical analysis

Continuous variables are presented as mean ± standard deviation (SD) and median (25th–75th percentile) for normal and non-normal distribution, respectively, and categorical data are summarized as frequencies (percentages). Normality of distribution was assessed with the Kolmogorov–Smirnov test in continuous variables. We applied logarithmic transformations to continuous variables showing a non-normal distribution. Differences between groups for continuous variables were analyzed by Student t test. For categorical variables, we evaluated the differences between groups with a chi-square test. Correlation between factors was analyzed by Pearson and Spearman correlation test when appropriate. The diagnostic value of SPP1 was calculated by constructing a receiver-operating characteristic (ROC) curve, and the optimal cutoff threshold was determined by Youden’s index. We constructed multivariable logistic regression models to assess the independent determinants of vulnerable plaque. Independent determinants for vulnerable plaque, including SPP1 level, gender, age, body mass index (BMI), smoke, diabetes mellitus, total to HDL cholesterol, estimated glomerular filtration rate (eGFR), high-sensitivity C-reactive protein (hsCRP), SYNTAX score, Gensini score and plaque burden were incorporated into multivariable logistic regression analyses. In the multivariate analysis, three models were developed. In model I, we included all conventional risk factors along with SPP1 level. Subsequently, in model 2, we added total-to-HDL cholesterol ratio, estimated glomerular filtration rate, and high-sensitivity C-reactive protein. In model 3, we further incorporated SYNTAX score, Gensini score, and plaque burden. All analyses were performed using 2-tailed tests with an overall significance level (alpha) of 0.05, and all tests were performed with SPSS 25.0 for Windows (SPSS, Inc., Chicago, IL, USA).

## 3 Results

### 3.1 Data preprocessing of the single-cell RNA sequencing

**Figure 1:**
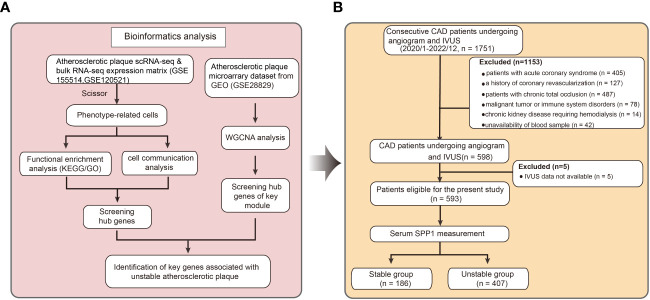
Flowchart describing the schematic overview of the current study design. **(A)** The microarray data, single cell and bulk RNA-seq atherosclerotic plaque data were downloaded from Gene Expression Omnibus. Key genes associated with unstable plaque were identified by bioinformatics analysis. **(B)** Clinical patient data were gathered and serum level of Secreted Phosphoprotein 1 were tested based on previous analysis. scRNA-seq indicates single-cell RNA sequencing; RNA-seq, RNA sequencing; KEGG, Kyoto Encyclopedia of Genes and Genomes; GO, Gene Ontology; CAD, coronary artery disease; IVUS, Intravascular Ultrasound; SPP1, Secreted Phosphoprotein 1.

**Figure 2:**
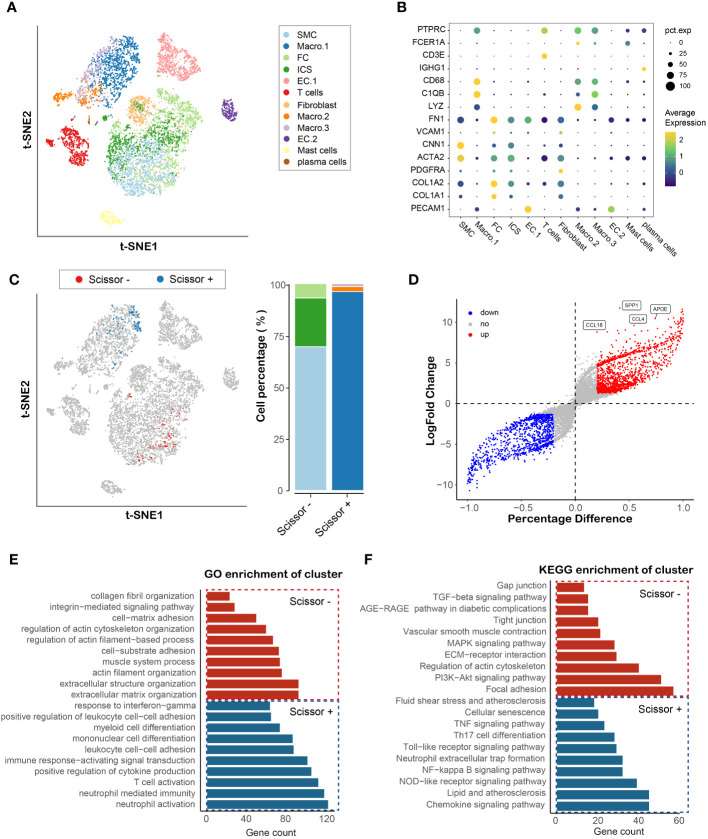
Identification and functional enrichment analysis of cell cluster associated with plaque vulnerability. **(A)** TSNE plot of atherosclerotic plaque from three human samples, showing 12 subpopulations in different colors. **(B)** Dot plot of cluster-identifying marker genes. **(C)** TSNE visualization of the Scissor-selected cells. The blue and red dots are Scissor+ (unstable plaque) and Scissor- (stable plaque) cells, respectively **(D)** Volcano plot of differential gene expressions in Scissor+ cells versus Scissor- cells. **(E, F)** Hallmark gene ontology (GO) and KEGG analysis between Scissor+ cells and Scissor- cells.

To identify potential cell types and biochemical markers associated with the vulnerable plaque, the flowchart of this research was set up and shown in **Figure 1A**. We downloaded the GSE155514 dataset containing three human plaque samples. The R package Seurat was utilized for data preprocessing. After a similar data preprocessing (**Supplementary Figures 1A, B**), we integrated three samples using the R package Harmony (**Supplementary Figure 1D**). We visualized the top 10 highly variable genes in **Supplementary Figure 1C**. A total of 4519 cells were identified after quality control. We then annotated the cell clusters based on previous studies and R package SingleR, and visualized with T-SNE plot. Overall, we identified twelve cell clusters on the basis of their expression genes levels, including six non-immune clusters and six leukocyte clusters (**Figure 2A**). The former included three endothelial cell clusters, one smooth muscle cell cluster, one fibroblast cell cluster, one fibrochondrocyte cluster and one intermediate cell state cluster (between SMC and FC), whereas the latter included one T cell cluster, one plasma cell cluster, one mast cell cluster and three macrophage cell clusters. Markers for defining these cell clusters were shown in **Figure 2B**.

### 3.2 Identifiers identification of phenotype-associated cell type and functional enrichment analysis of hub genes

To further the understanding of the role of cells in plaques in the vulnerable plaque development, we integrated bulk RNA-seq and single-cell sequencing data using R package Scissor. By using bulk expression matrix and clinical information, we successfully identified 264 phenotype-associated cells, of which 165 were Scissor+ (cells were positively associated with vulnerable plaque (**Figure 2C**). Macrophage.1 accounted for the majority, while a small percentage of Macrophage.2 and Macrophage.3 were present. Vascular Smooth muscle cells, intermediate cell state and fibrochondrocytes formed Scissor- cell cluster. To uncover the underlying transcriptional patterns of the identified cells linked to vulnerable plaque, we compared the gene expression between Scissor+ cell cluster and Scissor- cell cluster. In total, 1240 significantly upregulated genes and 1055 significantly downregulated genes were detected (**Figure 2D**). SPP1 showed the most significant change in the differential expression genes, indicating its potential role in the development of vulnerable plaque associated with macrophage. GO enrichment analysis revealed that the Scissor+ cell cluster was mainly involved in inflammatory process, such as neutrophil activation, t cell activation, cytokine production and leukocyte cell adhesion or differentiation (**Figure 2E**). KEGG enrichment analysis further confirmed the upregulation of the inflammation related signaling pathways (NF-κB, Nod-like receptor, TNF signaling pathways), which was consistent with the molecular function of macrophages. In addition, the Scissor- cell cluster was found to be mainly involved in extracellular matrix organization and muscle system process. Pathway enrichment analysis indicated that PI3K-akt, MAPK and TGF-beta signaling pathways were markedly enriched and associated with the Scissor- cell cluster (**Figure 2F**). Therefore, our integration analysis of single cell RNA and RNA sequencing data by Scissor identified the cell subpopulation that are most highly correlated with the development of vulnerable plaque and emphasized the critical function of the Scissor+/- cell cluster in the vulnerable plaque formation.

### 3.3 Cell-cell communication network construction

**Figure 3:**
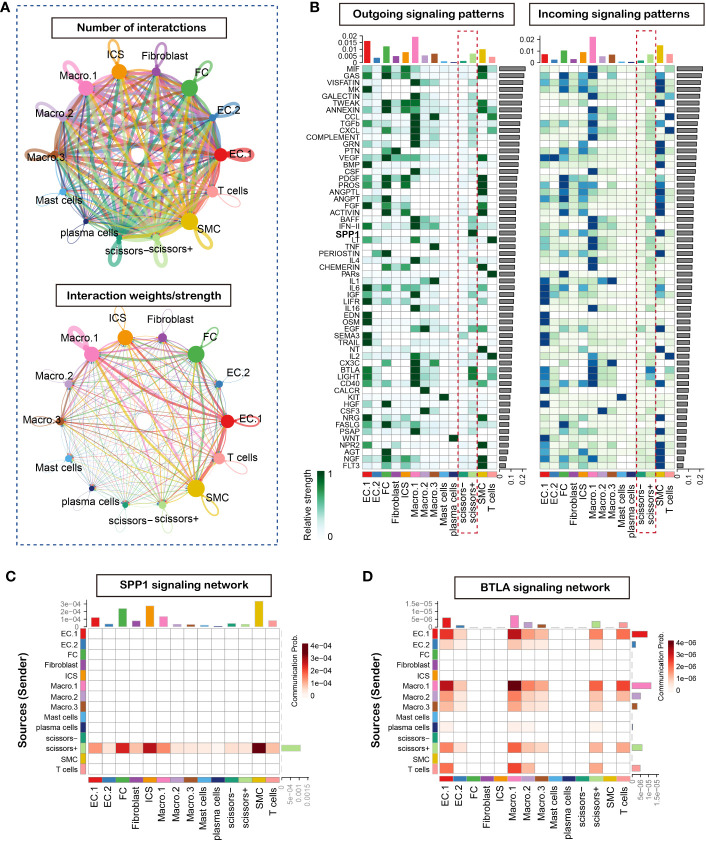
CellChat analysis of the communications between cells in atherosclerosis plaque. **(A)** Circle plot of number and strength of interactions in atherosclerotic plaque from three human samples, Circular compartments representing subclusters with weighted sizes. **(B)** Heatmap shows the relative importance outcoming and incoming signal network of each cell group based on the network centrality analysis **(C)** Heatmap of SPP1 signaling network. **(D)** Heatmap of BTLA signaling network.

Cell-cell communication is intricate and plays a pivotal role in vulnerable plaque formation. To delineate the cell communication of cell clusters especially phenotype-related, we analyzed and inferred the communication network using the R package CellChat, which contained a manually curated ligand-receptor database called CellChatDB and allowed user-assigned cell labels input. We used the circle plots to show the number and strength of interactions in the total cell clusters, and results suggested complex interactions among these cell clusters (**Figure 3A**). To further the understanding of the input and output signaling patterns among these cell clusters, heatmap was utilized to describe potential signaling pathways. Our result revealed that Scissor+ cell cluster rather than Scissor- cell cluster functioned as an activated signaling sender and receiver (**Figure 3B**). Of note, SPP1 signaling was found to be the most prominent output signaling pathway (**Figure 3C**) and BTLA signaling as the most significant input signaling pathway (**Figure 3D**). Furthermore, we found that the Scissor+ cell cluster was as the only cluster to send SPP1 signaling to interact with multiple cell types, including non-immune cells (endothelial cells, fibrochondrocytes, fibroblasts, smooth muscle cells and ICS cells) and T cells. Importantly, since the ICS cell cluster represented the intermedia status between FC and VSMC, our results indicated that SPP1 signaling may significantly influence the smooth muscle cell phenotype switching. Taken together, these observations highlighted the important function of SPP1 signaling of phenotype-related cells in the plaque instability.

### 3.4 Identification of key modules and hub genes through WGCNA analysis

**Figure 4:**
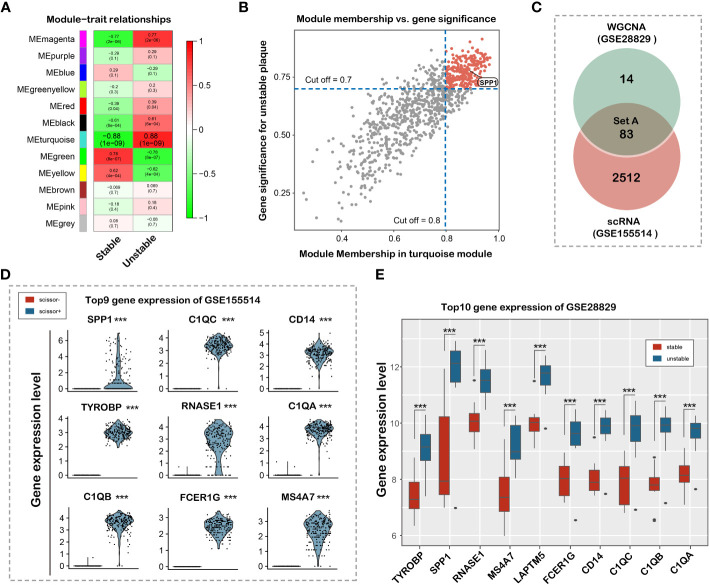
Identification and validation of hub genes associated with plaque vulnerability. **(A)** Heat map of the correlation between clinical traits, including Stable and Unstable. Each column corresponds to a clinical trait, and each row corresponds to a module. Each box contains the corresponding correlation coefficient and *P* value. Green represents negative correlation, and red represents positive correlation. **(B)** Correlation between MM of modules of interest and GS with clinical traits. Scatterplot of GS for Unstable vs MM in the turquoise module. **(C)** Venn diagram showing 83 overlapped hub genes **(D)** Violin plot of top 9 differential gene expression between Scissor+ and Scissor- cell cluster in GSE155514 dataset. **(E)** Box plot of top 10 differential gene expression in GSE28829 dataset.

We applied WGCNA methods to explore the co-expression genes of the disease using the expression profile and clinical information in GSE28829 dataset. The top 5000 of genes with high expression variance were selected for analysis. One specimen (GSM714096) was removed before constructing the weighted co-expression network for its incorrect clustering (**Supplementary Figure 2**). We found that when the power value was set to 18, the scale independence was >0.90 and the mean connectivity was higher (**Supplementary Figures 3A–E**). Next, we established the co-expression modules for further analysis using a hierarchical clustering tree. 12 modules were identified by their unique color and the number of genes in these modules ranged from 93 to 962 (**Supplementary Figure 4**). The gray module represented genes without similar expression patterns and was eliminated for further analysis. As shown in the results, genes in the turquoise module were most significantly associated with the occurrence of vulnerable plaque (**Figure 4A**). A scatterplot showed that 97 co-expression genes were highly associated with vulnerable plaque trait in turquoise module with module membership >0.8 and gene significance >0.7 (**Figure 4B**). 83 hub genes were obtained by combining co-expression genes and DEGs in single cell RNA-sequencing dataset (**Figure 4C**). The top differential genes in the GSE155514 and GSE28829 dataset were shown in **Figures 4D, E**, respectively. We found that SPP1 served as the most prominent gene based on DEGs and cell-cell communication analysis. In summary, we identified and validated that SPP1 in the turquoise module as the most significant gene for the occurrence of vulnerable plaque.

### 3.5 Hub gene validation in an independent patient cohort

**Figure 5:**
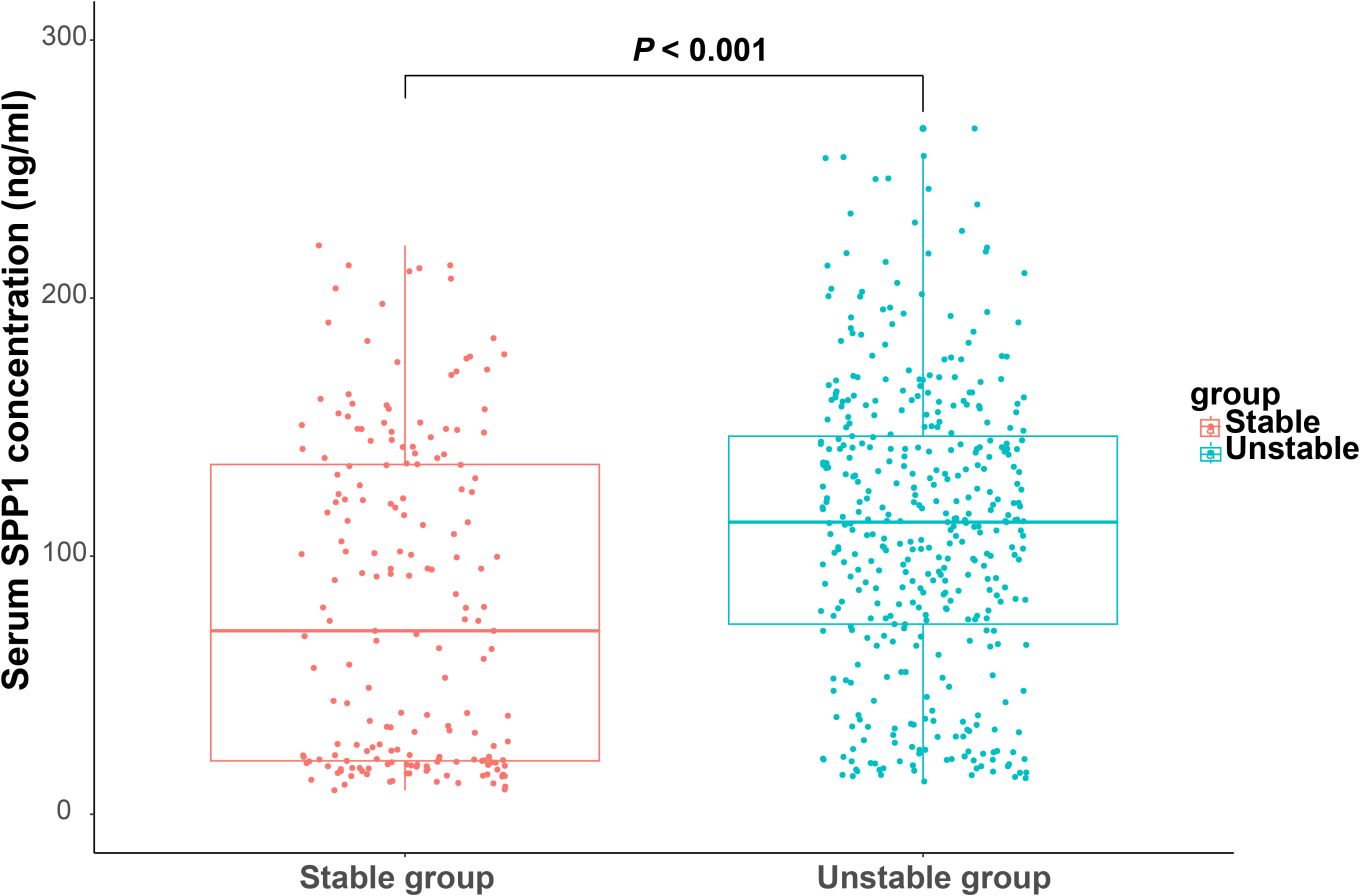
Serum secreted phosphoprotein 1 (SPP1) in coronary artery disease patients with stable and unstable plaque. Boxplot of serum SPP1 in patients with stable and unstable plaque.

To explore the potential role of SPP1 in clinical practice, we enrolled patients undergoing coronary angiography and IVUS from the database of the Shanghai RuiJin Hospital Percutaneous Coronary Intervention Outcomes Program. A total of 593 patients met the inclusion criteria (**Figure 1B**). Patients with unstable plaque identified by IVUS had higher SYNTAX (12.38 ± 7.14 versus 9.17 ± 5.16; *P* < 0.001), higher Gensini score (25.13 ± 17.41versus 16.82 ± 11.79; *P* < 0.001), elder age (65.24 ± 10.31 versus 61.98 ± 10.09; *P* < 0.001) and exhibited higher serum levels of SPP1(113.21 [73.6 - 147.70] ng/ml versus 71.08 [20.64 - 135.68] ng/ml; *P* < 0.001) in compassion with those with stable plaque (**Figure 5**). No significant differences were detected between the two groups with respect to gender, body mass index, history of cigarette smoking, hypertension, prior myocardial infarction and diabetes, blood pressure, renal function, lipid profiles, plaque burden and medical treatments (all *P* > 0.05) (**Table 1**). In the correlation analyses, no significant correlation was found between serum SPP1 levels and plaque burden (r = -0.010, *P* = 0.804) or minimal lumen area (r = -0.501, *P* = 0.219).

**Table 1:** Baseline characteristics of patients with stable and unstable plaque.

Demographic characteristics and clinical assessments	Stable group (n = 186)	Unstable group (n = 407)	P value
Male, n (%)	117 (62.9)	285 (70.0)	0.085
Age, years	61.98 ± 10.09	65.24 ± 10.31	<0.001
Body Mass Index, Kg/m^2^	24.71 ± 2.97	24.83 ± 3.14	0.648
Cigarette smoking, n (%)	58 (31.2)	129 (31.7)	0.901
Hypertension, n (%)	127 (68.3)	287 (70.5)	0.582
Systolic blood pressure, mm Hg	138.78 ± 18.81	139.68 ± 19.47	0.596
Diastolic blood pressure, mm Hg	78.36 ± 10.97	76.99 ± 10.99	0.160
Previous myocardial infarction, n (%)	5 (2.7)	17 (4.2)	0.373
Diabetes, n (%)	45 (24.2)	124 (30.5)	0.116
Laboratory measurements			
Fasting blood glucose, mmol/L	5.80 ± 1.64	6.17 ± 3.89	0.225
HbA1c, %	6.08 ± 0.99	6.27 ± 1.09	0.047
Serum creatinine, μmol/L	79.43 ± 28.38	81.88 ± 41.73	0.404
Serum uric acid, μmol/L	334.60 ± 83.39	348.08 ± 88.52	0.081
eGFR, mL/min/1.73m^2^	85.56 ± 15.99	82.19 ± 16.72	0.022
NT-proBNP, pg/mL	218.76 ± 998.29	215.75 ± 693.79	0.967
Triglyceride, mmol/L	1.57 ± 0.82	1.63 ± 1.02	0.482
Total cholesterol, mmol/L	4.01 ± 1.06	4.09 ± 1.14	0.385
HDL cholesterol, mmol/L	1.13 ± 0.26	1.16 ± 0.34	0.202
LDL cholesterol, mmol/L	2.33 ± 0.90	2.36 ± 0.94	0.710
Apolipoprotein A, g/L	1.25 ± 0.21	1.26 ± 0.21	0.445
Apolipoprotein B, g/L	0.77 ± 0.23	0.79 ± 0.26	0.234
Lipoprotein (a), g/L	0.26 ± 0.32	0.27 ± 0.31	0.680
hsCRP, mg/L	0.91 (0.36 - 2.00)	1.00 (0.48 - 2.30)	0.108
SPP1, ng/ml	71.08 (20.64 - 135.68)	113.21 (73.65 - 147.70)	<0.001
Quantification of coronary lesions			
Gensini score	16.82 ± 11.79	25.13 ± 17.41	<0.001
SYNTAX score	9.17 ± 5.16	12.38 ± 7.14	<0.001
Minimum lumen area, mm^2^	3.99 ± 1.88	3.67 ± 2.16	0.090
Plaque burden, %	69.60 (60.31 - 75.46)	72.20 (66.37 - 77.71)	<0.001
Medication, n (%)			
ACE inhibitors/ARBs/ARNI	68 (36.8)	173 (42.5)	0.187
β-blockers	59 (31.7)	110 (27.0)	0.240
Calcium channel blockers	67 (36.0)	126 (310)	0.222
Statins	121 (65.1)	289 (71.0)	0.145

Values are given as mean ± SD, median (25th–75th percentile), or number (percentage). HbA1c, glycated hemoglobin; eGFR, estimated glomerular filtration rate; NT-proBNP, N-terminal pro-B-type natriuretic peptide; HDL, high‐density lipoprotein; LDL, low‐density lipoprotein. hs‐CRP, high‐sensitivity C‐reactive protein; SPP1, Secreted Phosphoprotein 1; ACEI indicates angiotensin‐converting enzyme inhibitor; ARB, angiotonin receptor blocker; ARNI, indicates angiotensin receptor neprilysin Inhibitor.

**Figure 6:**
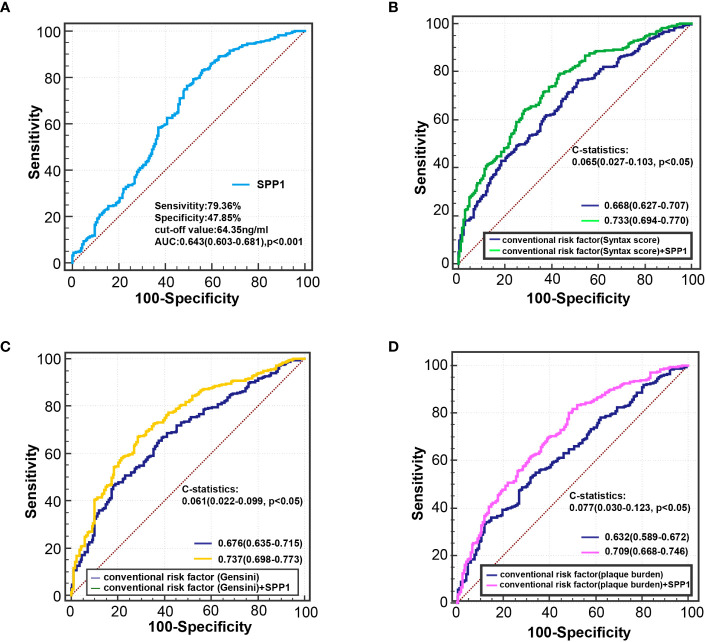
Receiver-operating characteristic curve analysis for identifying vulnerable plaque. **(A)** ROC curve of serum SPP1 for diagnosing vulnerable plaque. **(B)** ROC curve derived from regression model 3a for detecting vulnerable plaque. conventional risk factors: age, sex, body mass index, smoking habits, hypertension, body mass index, diabetes, estimated glomerular filtration rate, Log hsCRP, Syntax score. **(C)** ROC curve derived from regression model 3b for detecting vulnerable plaque. conventional risk factors: age, sex, body mass index, smoking habits, hypertension, body mass index, diabetes, estimated glomerular filtration rate, Log hsCRP, Gensini score. **(D)** ROC curve derived from regression model 3c for detecting vulnerable plaque. conventional risk factors: age, sex, body mass index, smoking habits, hypertension, body mass index, diabetes, estimated glomerular filtration rate, Log hsCRP, plaque burden. hsCRP indicates high-sensitivity C-reactive protein; and ROC, receiver operating characteristic.

**Figure 7:**
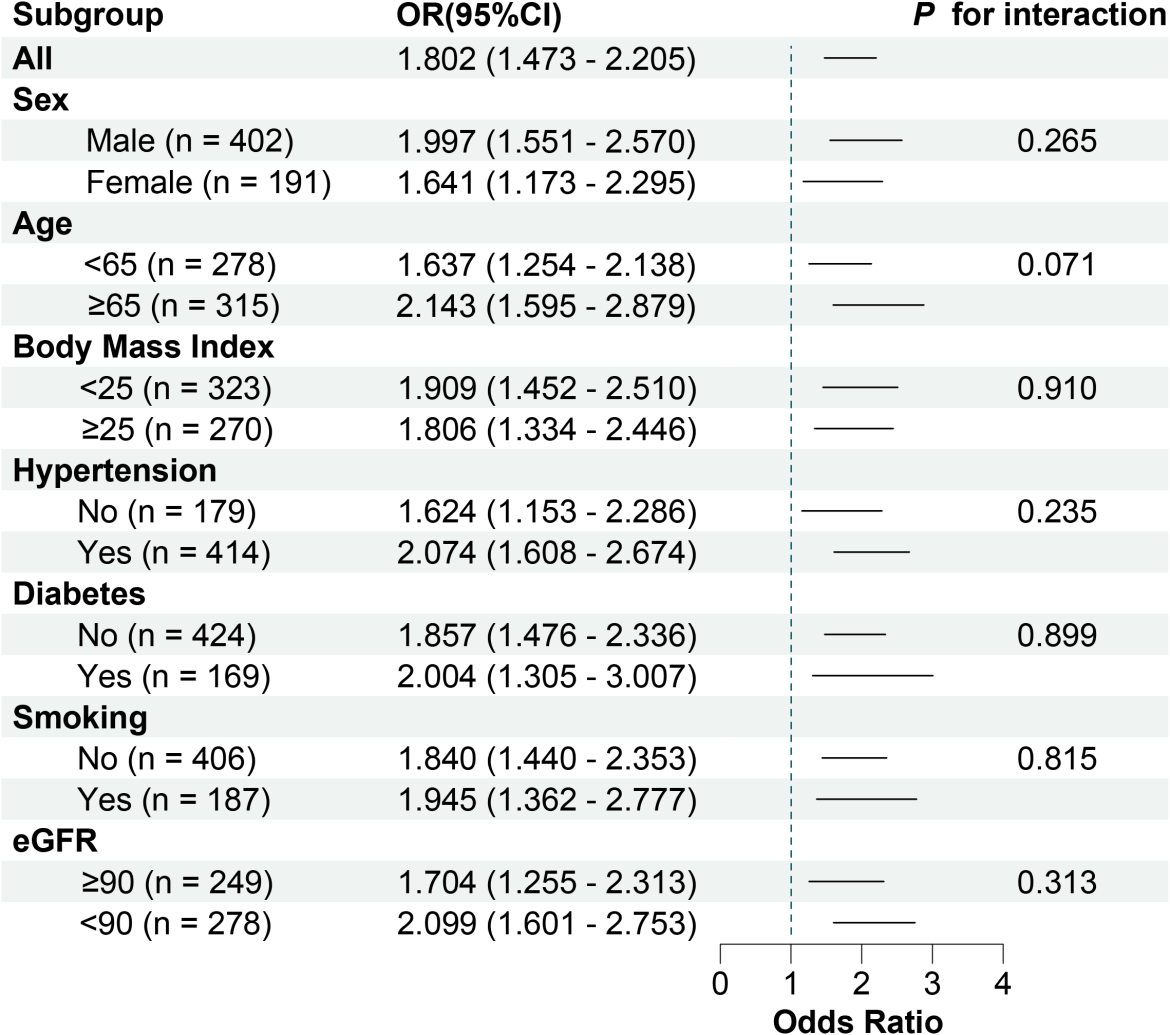
Forest plot of adjusted ORs in subgroups. eGFR, estimated glomerular filtration rate; and ORs, odd ratio.

Receiver operating characteristic curve analysis showed that the area under the curve was 0.643 (95% CI, 0.603 - 0.681; *P* < 0.001) for serum SPP1 in predicting of patients with unstable plaque, with an optimal cutoff point of 64.354 ng/ml (sensitivity = 79.36%, and specificity = 47.85%) (**Figure 6A**). Besides, receiver operating characteristic curve in models adjusted for conventional risk factors showed that the addition of SPP1 effectively elevated the AUC (**Figures 6B-D**). Inclusion of SPP1 in the model considering Gensini score as conventional risk factor showed the best AUC (AUC = 0.737, 95% CI, 0.697 - 0.773; *P* < 0.001), with a significant C statistics 0.061(95%CI, 0.022 - 0.099; *P* = 0.002) compared with conventional risk factor model. In the subgroup analysis, we confirmed that the results were consistent in all subgroup patients and higher level of serum SPP1 was indicative of vulnerable plaques (**Figure 7**).

We performed logistic regression analyses to determine the association between SPP1 and patients with unstable plaque. In model 1 (**Table 2**), major risk factors including male, age, BMI, hypertension, smoking, and diabetes were included. SPP1 level was divided into 2 groups based on the cutoff value from receiver operating characteristic curve. Group 1 indicated patients with serum SPP1 level less than 64.354 (ng/ml), and Group 2 indicated serum SPP1 level equaled to or larger than 64.354. The result showed that high level of SPP1 (OR = 3.354, 95% CI, 2.289 – 4.915; *P* < 0.001) was significantly associated with unstable plaque. In model 2, total-to-HDL cholesterol ratio, eGFR and Log hsCRP were included together with abovementioned factors in model 1. High level of SPP1 remained a significant independent risk factor of unstable plaque (OR =3.390, 95% CI, 2.236- 5.139; *P* < 0.001). In model 3a, 3b and 3c, when SYNTAX score, Gensini score and plaque burden was further included on the basis of model 2, respectively, high level of SPP1 still remained significant to be independent determinants of unstable plaque. (OR_Model 3a _= 3.344, 95% CI, 2.188 - 5.110; OR_Model 3b_ = 3.173, 95% CI, 2.078 – 4.843; and OR_Model 3c_ = 3.469, 95% CI, 2.282 - 5.273; all *P* < 0.001).

**Table 2:** Multivariate logistic regression analyses for coronary artery disease in patients with stable and unstable plaque.

Variables	Model 1		Model 2		Model 3a		Model 3b		Model 3c	
	OR (95% CI)	*P* value	OR (95% CI)	*P* value	OR (95% CI)	*P* value	OR (95% CI)	*P* value	OR (95% CI)	*P* value
SPP1(Group 1)										
SPP1(Group 2)	3.354 (2.289 - 4.915)	<0.001	3.390 (2.236 - 5.139)	<0.001	3.344 (2.188 - 5.110)	<0.001	3.173 (2.078 - 4.843)	<0.001	3.469 (2.282 - 5.273)	<0.001
Male	1.664 (1.068 - 2.593)	0.025	1.666 (1.038 - 2.673)	0.034	1.460 (0.899 - 2.373)	0.126	1.415 (0.870 - 2.302)	0.162	1.706 (1.058 - 2.748)	0.028
Age	1.034 (1.015 - 1.054)	<0.001	1.042 (1.017 - 1.067)	0.001	1.037 (1.012 - 1.063)	0.003	1.036 (1.011 - 1.061)	0.005	1.041 (1.016 - 1.066)	0.001
Body Mass Index	1.019 (0.958 - 1.083)	0.556	1.021 (0.955 - 1.091)	0.544	1.029 (0.961 - 1.102)	0.417	1.027 (0.959 - 1.100)	0.447	1.023 (0.956 - 1.094)	0.510
Smoke	0.883 (0.561 - 1.388)	0.588	0.882 (0.546 - 1.420)	0.609	0.848 (0.520 - 1.383)	0.509	0.871 (0.534 - 1.421)	0.580	0.901 (0.554 - 1.463)	0.673
Hypertension	0.896 (0.596 - 1.345)	0.596	0.911 (0.591 - 1.404)	0.672	0.859 (0.552 - 1.335)	0.498	0.857 (0.552 - 1.330)	0.491	0.938 (0.606 - 1.450)	0.773
Diabetes	1.220 (0.802 - 1.856)	0.354	1.316 (0.848 - 2.042)	0.220	1.174 (0.750 - 1.840)	0.483	1.132 (0.721 - 1.778)	0.591	1.295 (0.832 - 2.017)	0.252
Total-to-HDL cholesterol			1.000 (0.976 - 1.026)	0.976	1.001 (0.977 - 1.027)	0.908	1.002 (0.975 - 1.030)	0.895	1.000 (0.976 - 1.026)	0.975
eGFR			0.999 (0.985 - 1.013)	0.902	0.998 (0.984 - 1.013)	0.801	0.998 (0.984 - 1.012)	0.790	0.999 (0.984 - 1.013)	0.842
Log hsCRP			0.995 (0.887 - 1.115)	0.927	0.987 (0.879 - 1.108)	0.820	0.987 (0.879 - 1.108)	0.825	0.994 (0.886 - 1.115)	0.922
SYNTAX score					1.076 (1.039 - 1.115)	<0.001				
Gensini score							1.033 (1.016 - 1.049)	<0.001		
Plaque burden									1.449 (0.627 – 3.349)	0.386

Model 1, adjustment for age, sex, body mass index, smoke, hypertension and diabetes; Model 2, additional adjustment for Total-to-HDL cholesterol, Log hypersensitive C-reactive protein, and estimated glomerular filtration rate; Model 3a, additional adjustment for SYNTAX score; Model 3b, additional adjustment for Gensini score; Model 3c, additional adjustment for plaque burden. HbA1c indicated glycated hemoglobin; eGFR, estimated glomerular filtration rate; HDL, high‐density lipoprotein; hs‐CRP, high‐sensitivity C‐reactive protein; SPP1, Secreted Phosphoprotein 1.

## 4 Discussion

In this study, we integrated single-cell transcriptome data from three human plaques and constructed a specific cell-cell network by combining single-cell and RNA-seq transcriptome data using the Scissor algorithm. Our network analysis suggested that SPP1 signaling (inflammatory macrophages as the main signal sender) acted as an important role in the vulnerable plaque formation and SPP1 as the hub gene associated with vulnerable plaque. Our study also demonstrated that elevated serum SPP1 level was an independent predictive factor in patients with vulnerable plaques.

Atherosclerosis featured a highly dynamic plasticity, during which macrophages were subjected to multiple microenvironmental signals, thus influencing its polarization and activation process. After analyzing atherosclerotic plaque scRNA-seq (GSE155514) and bulk RNA-seq expression matrix (GSE155514) using the scissor algorithm, we identified mixed macrophage clusters that were significantly associated with plaque instability. Functional enrichment analysis revealed that signaling pathways such as inflammation-related TNF signaling, Nod-like receptors, or NF-κB were highly enriched in the modified cell cluster, suggesting that this macrophage subpopulation was predominantly pro-inflammatory macrophages. A large amount of evidence shows that the number of pro-inflammatory macrophages is positively correlated with the stability of atherosclerotic plaques (39–41), which also confirms that the single-cell analysis of this study is credible and reasonable. To identify the factors most correlated with plaque stability, we used WGCNA to analyze another independent atherosclerotic plaque dataset (GSE28829). After intersecting the hub genes screened out by the scissor algorithm and WGCNA, the most prominent DEG, SPP1, was determined (**Figure 4**). SPP1, also known as osteopontin, is a type of secreted glycoprotein with similar structure and function to the matricelluar protein (42). SPP1 expression was found to be mainly driven by proinflammatory cytokines including IL-1β, TNFα and IL-6 (43). Previous studies have confirmed that SPP1 was involved in multiple macrophage biological processes, including adhesion (44), migration (45), chemotaxis (46), and polarization (47). Macrophages in murine SPP1 deletion model showed significantly impaired phagocytosis ability compared with those in normal murine model (48). Silencing SPP1 gene in APOE deficient mice had smaller atherosclerotic lesion sizes and inflammatory cell (especially macrophage) infiltration areas (49), indicating important chemotactic role that SPP1 plays in the regulation of proinflammatory macrophages. Consistent result have also been detected in abdominal aortic aneurysm model in ApoE^–/–^OPN^–/–^mice characterized by impaired leukocyte recruitment and cell migration (50). In addition, SPP1 knockdown also facilitated macrophage polarization toward the M2 subtype (51), generally considered to be the anti-inflammatory macrophage.

Cellular crosstalk is fundamental to the development of vulnerable plaques since intercellular communication drives many pathological processes including cell signaling transduction and cell differentiation. Our cell-cell communication analysis revealed that SPP1 signaling was the exclusive signaling from Scissor+ macrophage cluster. Besides, endothelial, FC and VSMC clusters were the major clusters responding to SPP1 signaling. One group reported that SPP1 could induce angiogenesis of endothelial cells through VEGF-dependent activation of signaling pathways such as PI3K/AKT and ERK1/2 *in vitro* (52). Moreover, SPP1 could promote endothelial cell migration through ERK1/2 activation (53). This phenomenon is consistent with the previous study that angiogenesis acted as a source of intraplaque hemorrhage—significantly associated with plaque instability (54). SPP1 was firstly postulated to influence the VSMCs migration process through integrin receptor (55). Recent study has confirmed that autocrine SPP1 was highly correlated with PDGF-mediated smooth muscle cell migration (56). In addition, SPP1 facilitated a more proliferative VSMC phenotype. Both migration and proliferation suggested a close relationship with intima thickness (57), which promotes macrophage infiltration and foam cell formation (58, 59). In addition, SPP1 expression represented a smooth muscle-derived foam cell phenotype (60) and was found to inhibit two specific VSMC differentiation makers (calponin and α-SM actin) (61). Therefore it has been commonly suggested that VSMC contributed largely to the different cell phenotypes in the development of atherosclerosis (62), although the underlying mechanism was still unknown.

The term “vulnerable plaque” was defined largely based on the concept that most future event-related atherosclerotic plaques in patients with acute coronary syndrome shared the characterization of angiographically mild stenosis (63, 64) and were often non-culprit lesions for the current condition (2) to highlight its pivotal role in potential clinical practice. The result of the first prospective natural cohort study suggested that such vulnerable plaques were associated with recurrent events and could be determined by gray-scale IVUS (4). Our results indicated that serum biomarker SPP1 was increased in patients with vulnerable plaque and served as a potential risk factor for plaque instability even after adjusting multiple variables. The receiver operating characteristic curve suggested that the addition of serum SPP1 level on the basis of traditional risk factor allowed a better detection of the vulnerable plaque. Previous studies revealed that SPP1 served as an effective prognostic or diagnostic biomarker in circulatory system diseases such as ischemic stroke (65), stable coronary artery disease (66, 67), heart failure (68, 69) and peripheral artery disease (17). Our studies clarified the potential vulnerable plaque landscape through integration of both single-cell and RNA transcriptome sequencing data and extended the information to a retrospective cohort likely to be representative of patients seen in clinical practice, ultimately demonstrating that serum SPP1 levels significantly contribute to the diagnostic of vulnerable plaque.

We acknowledge that there are limitations in our current findings. First, this study is a cross-sectional study with a small sample size, which prevents us from predicting long-term endpoints such as recurrent myocardial infarction or cardiovascular death. Second, the spatial resolution of gray scale IVUS does not suffice us to detect all crucial features of vulnerable plaque. For example, the histopathological definition of thin-cap fibroatheroma thickness was usually less than 65μm, thereby leading to the overestimation of the number of TCFA lesions. Third, although we applied rigorous data preprocessing, normalization, and batch correction techniques to harmonize the datasets as much as possible, the integration of bulk RNA sequencing data and single-cell RNA sequencing data from different datasets may introduce a potential source of confounding in our results. However, we only focused on the most robust and consistent findings across both datasets, and validated our results with independent clinical data. Finally, further molecular research is required to clarify the potential mechanism of SPP1 on plaque vulnerability.

## 5 Conclusions

In summary, our study demonstrated that SPP1 identified by integrating of single cell and RNA sequencing analysis is associated with plaque vulnerability. These observations may provide substantial insights for predicting vulnerable plaques in patients with coronary artery disease. However, further studies are warranted to elucidate the specific mechanism of SPP1 in the regulation of vulnerable plaque formation in patients with coronary artery disease.

## Data availability statement

The original contributions presented in the study are included in the article/**Supplementary Material**. Further inquiries can be directed to the corresponding authors.

## Ethics statement

The studies involving humans were approved by Ethics Committee of the Ruijin Hospital and Shanghai Jiao Tong University School of Medicine. The studies were conducted in accordance with the local legislation and institutional requirements. The participants provided their written informed consent to participate in this study.

## Author contributions

KH: Writing – original draft. SC: Writing – original draft. LY: Writing – review & editing. ZW: Writing – review & editing. QC: Writing – review & editing. X-QW: Writing – review & editing. F-FL: Writing – review & editing. JL: Writing – review & editing. YW: Writing – review & editing. LM: Writing – review & editing. WS: Writing – review & editing. RZ: Writing – review & editing. YS: Writing – review & editing. LL: Writing – review & editing. YD: Writing – original draft. FD: Writing – original draft.
