# Supplementary material for: Serum secreted phosphoprotein 1 level is associated with plaque vulnerability in patients with coronary artery disease
Source: Front Immunol. 2024 Feb 15;15:1285813. doi: 10.3389/fimmu.2024.1285813 (PMC10902157; doi:10.3389/fimmu.2024.1285813)
Supplement: Supplementary file 1 [file DataSheet_1.docx]

Supplementary Material

# Supplementary Figures


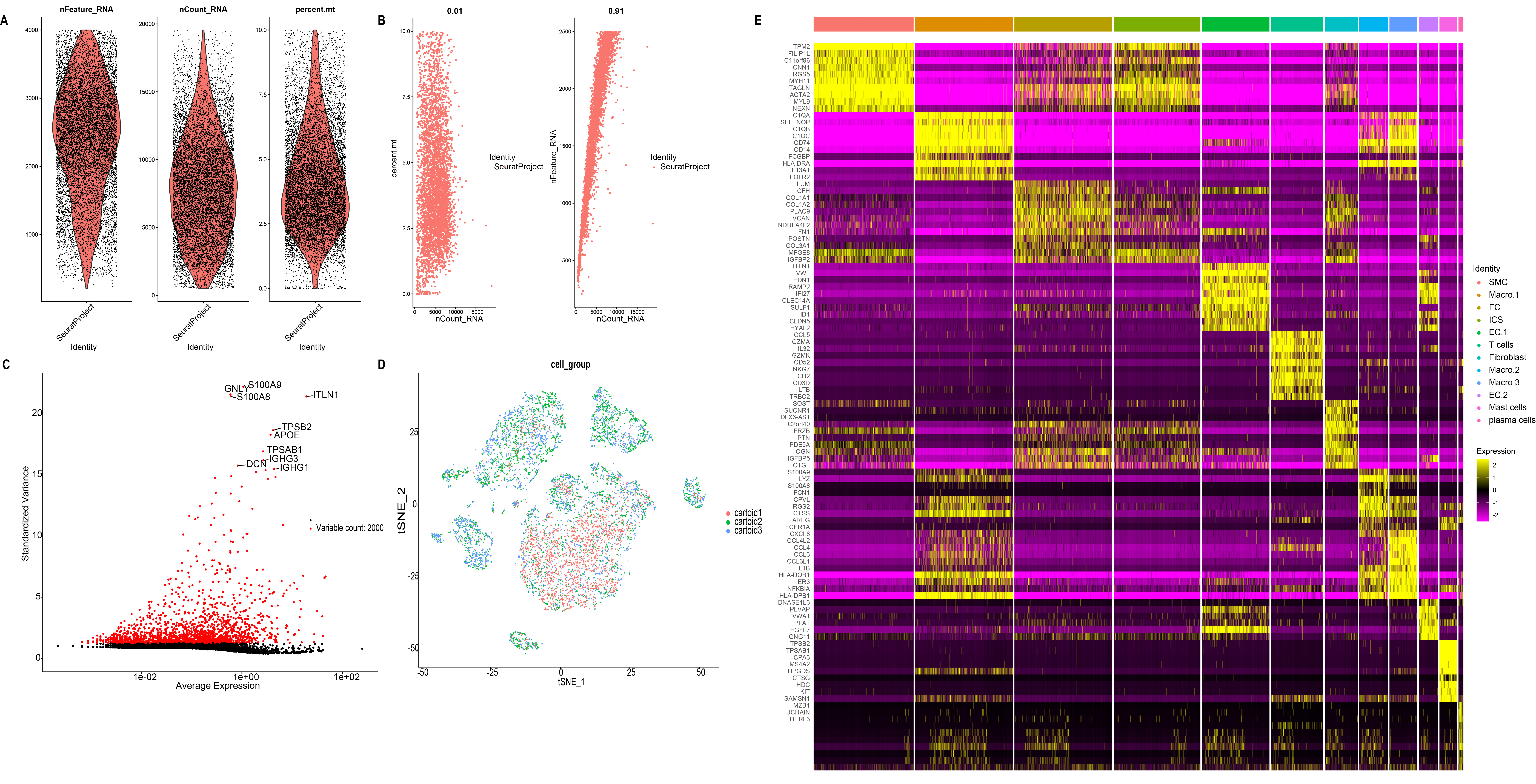


**Supplementary Figure 1 |** **Single-cell RNA-seq data quality control**(A) Violin plots showing quality control (analyses of nFeature counts, nCountRNA and mitochondrial genes) after combing 3 samples. (B) The numbers of detected genes were significantly related to the percentage of mitochondria and sequencing depth, with a Pearson’s correlation coefficient of 0.01, 0.91, respectively (C) The variance diagram shows 17818 corresponding genes throughout all cells from combined single cell dataset. The red dots represent highly variable genes, and the black dots represent non-variable genes. The top 10 most variable genes are marked in the plot. (D) TSNE plot of single cell dataset after integration with R package Harmony. (E) Heatmap of top 10 differential expression genes in each cell cluster. The colors from purple to yellow indicate the gene expression levels from low to high.


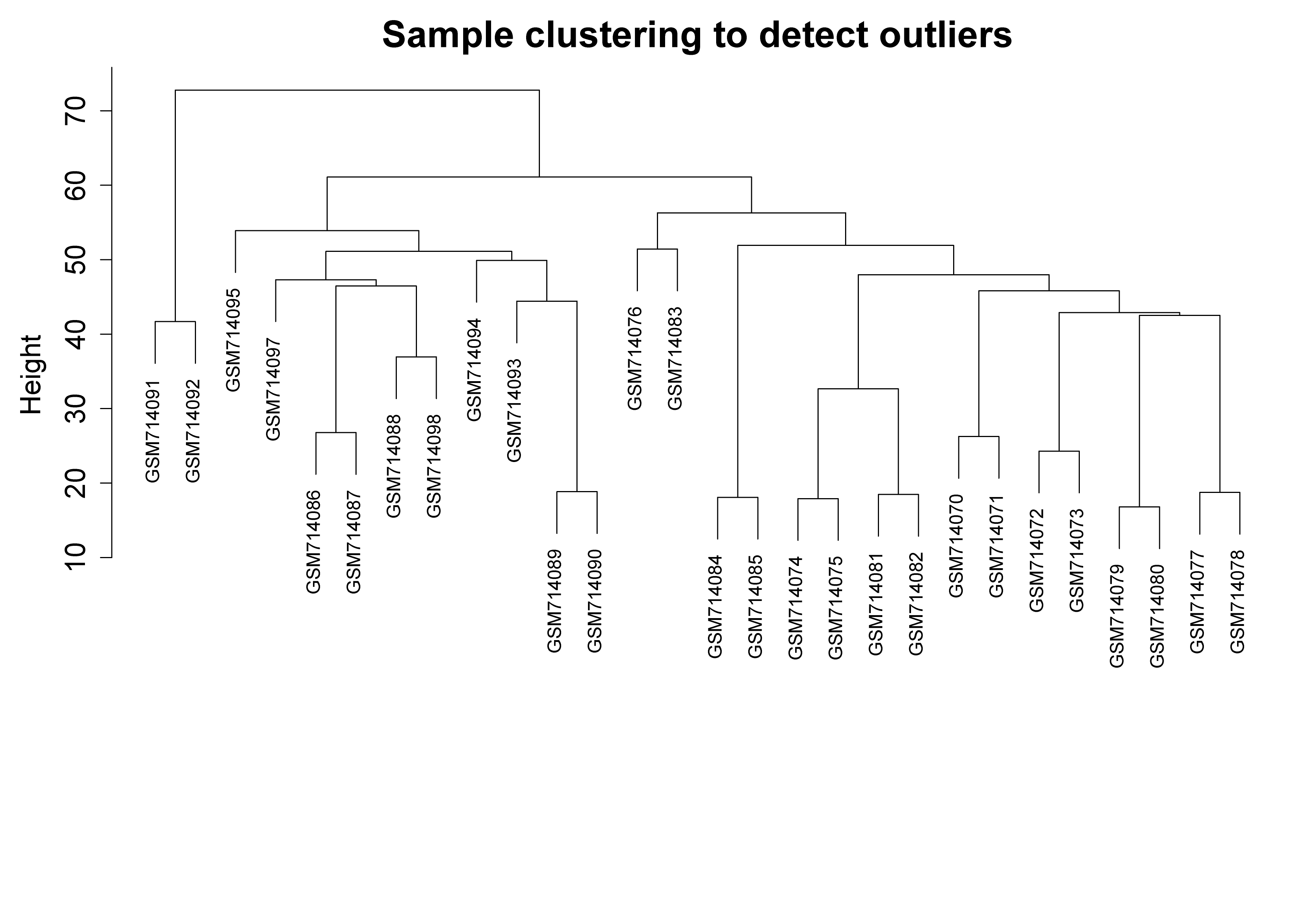


**Supplementary Figure 2 |** **Sample clustering to detect outliers**


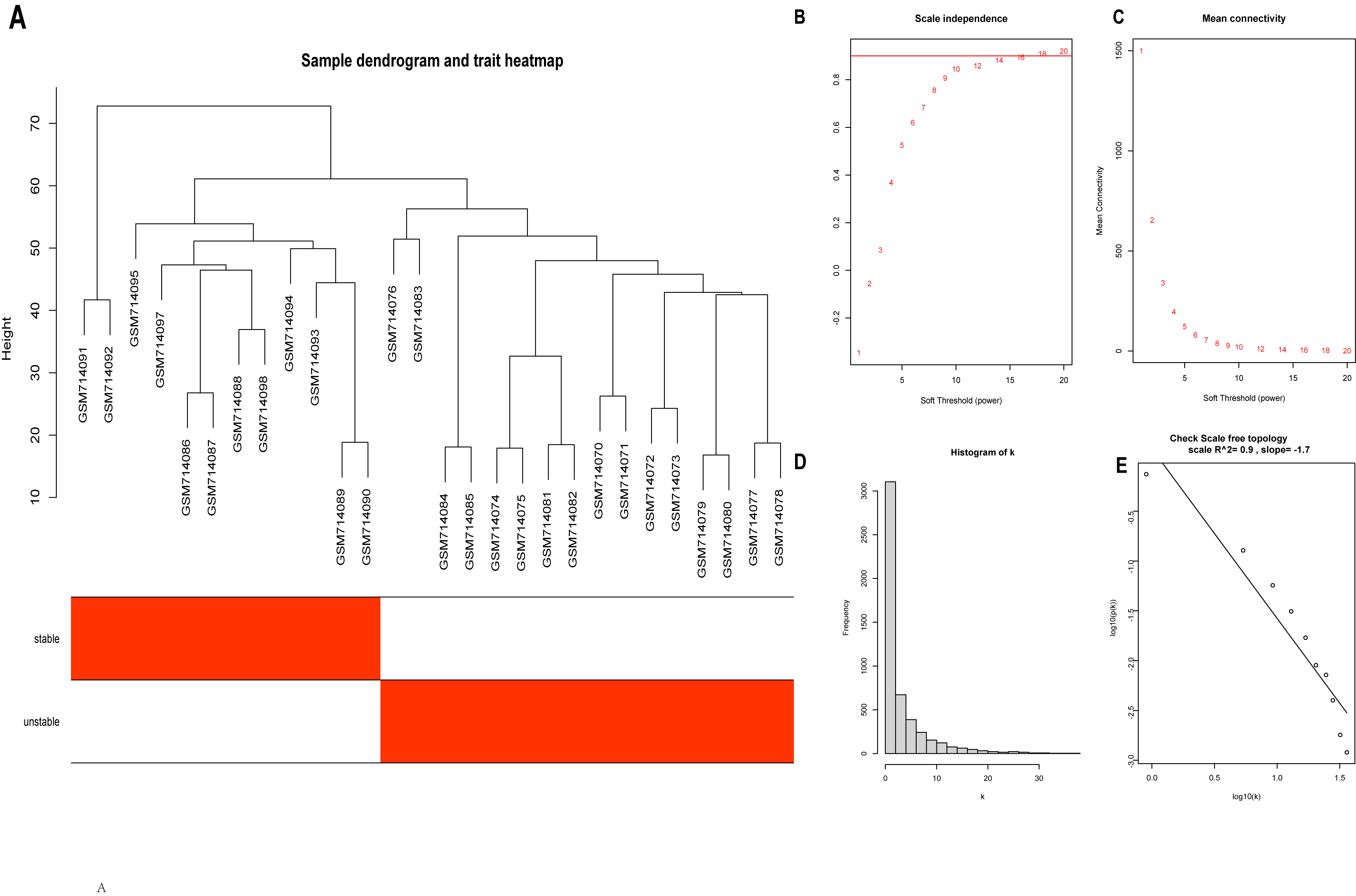


**Supplementary Figure 3 |** **Clustering of samples and determination of soft thresholding power** (A) Sample dendrogram and trait heatmap. The clustering analysis was based on the GSE28829 expression matrix, which contained of 12 stable and 16 unstable plaques. (B) Analysis of scale-independence index for various soft threshold powers. (C) Analysis of mean connectivity for various soft threshold powers. (D) The histogram of k of the samples. (E) The correlation coefficient between k and p (k) of the samples, and the R^2^ reached 0.9.


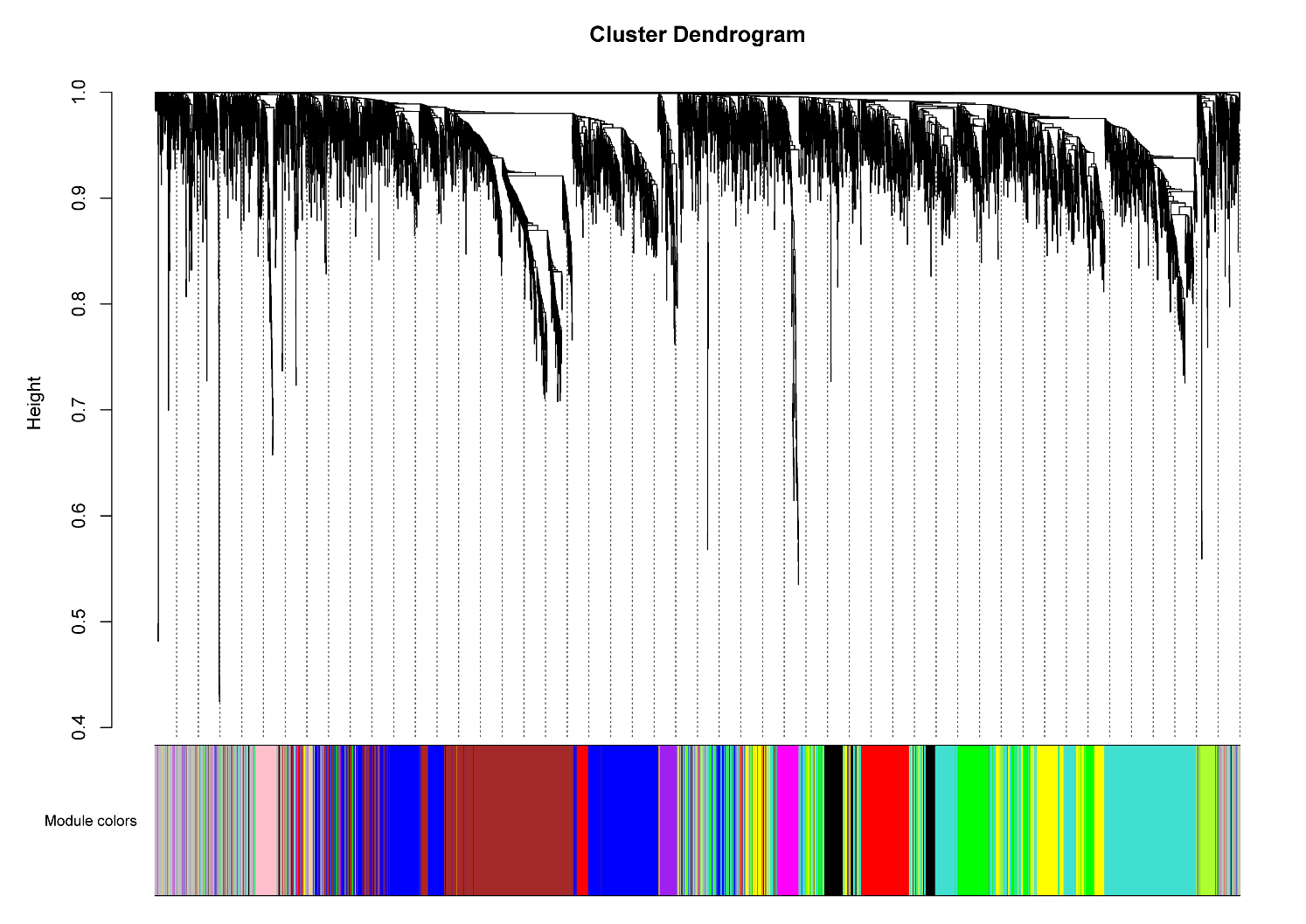


**Supplementary Figure 4** **| Construction of co-expression modules by the weighted gene co-expression network analysis (WGCNA) package**
